# Association between estimated glucose disposal rate and metabolic dysfunction-associated steatotic liver disease and dyslipidemia in US adults: a cross-sectional study

**DOI:** 10.3389/fnut.2025.1621074

**Published:** 2025-07-02

**Authors:** Deliang Liu, Xiaojun Lv, Wenwen Li, Hongsheng Dai, Yang Tan, Dandan Yang, Xiaoqian Zhang

**Affiliations:** ^1^Department of Gastroenterology, Affiliated Hospital of Shandong Second Medical University, Weifang, China; ^2^School of Clinical Medicine, Shandong Second Medical University, Weifang, China

**Keywords:** IR, eGDR, MASLD, lipid, NHANES

## Abstract

**Background:**

Metabolic dysfunction-associated steatotic liver disease (MASLD) is strongly associated with insulin resistance (IR). This study examined the relationship between estimated glucose disposal rate (eGDR), a simple IR indicator, and MASLD risk.

**Methods:**

Using NHANES 2017–2018 data (*n* = 3,957), MASLD was diagnosed by CAP ≥285 dB/m. eGDR was calculated from waist circumference, hypertension, and HbA1c.

**Results:**

Lower eGDR significantly predicted higher MASLD risk (OR = 0.396, *p* < 0.01) and elevated CA*p* values (*b* = −21.375, *p* < 0.01). It also correlated with unfavorable lipid profiles (lower HDL, higher triglycerides). Subgroup analyses showed consistent associations across age, sex, and diabetes status.

**Conclusion:**

Estimated glucose disposal rate was significantly associated with both MASLD and dyslipidemia. eGDR may serve as a useful indicator for identifying risk factors related to these metabolic disorders. Mediation analysis revealed that relative fat mass (RFM), (high-density lipoprotein cholesterol) HDL, triglyceride (TG), visceral adiposity index (VAI), and uric acid to HDL ratio (UHR) mediated the association between eGDR and MASLD, with respective proportions of 61.09, 6.79, 6.53, 9.85, and 12.9%.

## 1 Introduction

Metabolic dysfunction-associated steatotic liver disease (MASLD) has evolved into a public health issue all over the world. MASLD endangers approximately 25 to 30% of adults worldwide and is considered a multisystem disease (1, 2). It is not only associated with liver-specific complications, like liver fibrosis and cirrhosis, but also with a series of extrahepatic diseases, comprising cardiovascular disease, diabetes, and renal disease. In recent years (3), the incidence of MASLD has also been rapidly increasing (4) as the morbidity of chronic metabolic diseases such as diabetes, obesity, and hypertension continues to rise. Identifying emerging risk factors related to the occurrence and progression of MASLD is therefore essential (5). This in turn will help detect risky populations and establish effective prevention and intervention measures to address this increasingly serious health challenge.

Estimated glucose disposal rate (eGDR) is a validated clinical approach designed to evaluate insulin resistance (IR) in individuals, particularly in patients with diabetes. This method is derived from common clinical indicators, including hypertension, waist circumference (WC), and glycated hemoglobin (HbA1c) (6). This approach offers a relatively easy alternative over more complex procedures such as the hyperinsulinemic-euglycemic clamp technique (7), which is cost-effective and scalable and suitable for large-scale cohort studies. Studies have shown that eGDR is correlated with the risk of multiple metabolic and cardiovascular diseases, like fatty liver, coronary heart disease, and stroke (8). By providing a reliable assessment of insulin sensitivity, eGDR has become a vital tool in clinical practice and research to detect patients with a high risk of chronic health problems at an early stage (9).

The study aims to explore the correlation between eGDR and MASLD and to assess the potential influence of dyslipidemia on this relationship. Although eGDR has connections with multiple metabolic diseases, like IR, type 2 diabetes, and cardiovascular disease, its role in the prognosis of MASLD has not been completely clarified. Expanded research is necessary to reveal its clinical implications in this area completely.

## 2 Methods

### 2.1 Study population

**Figure 1:**
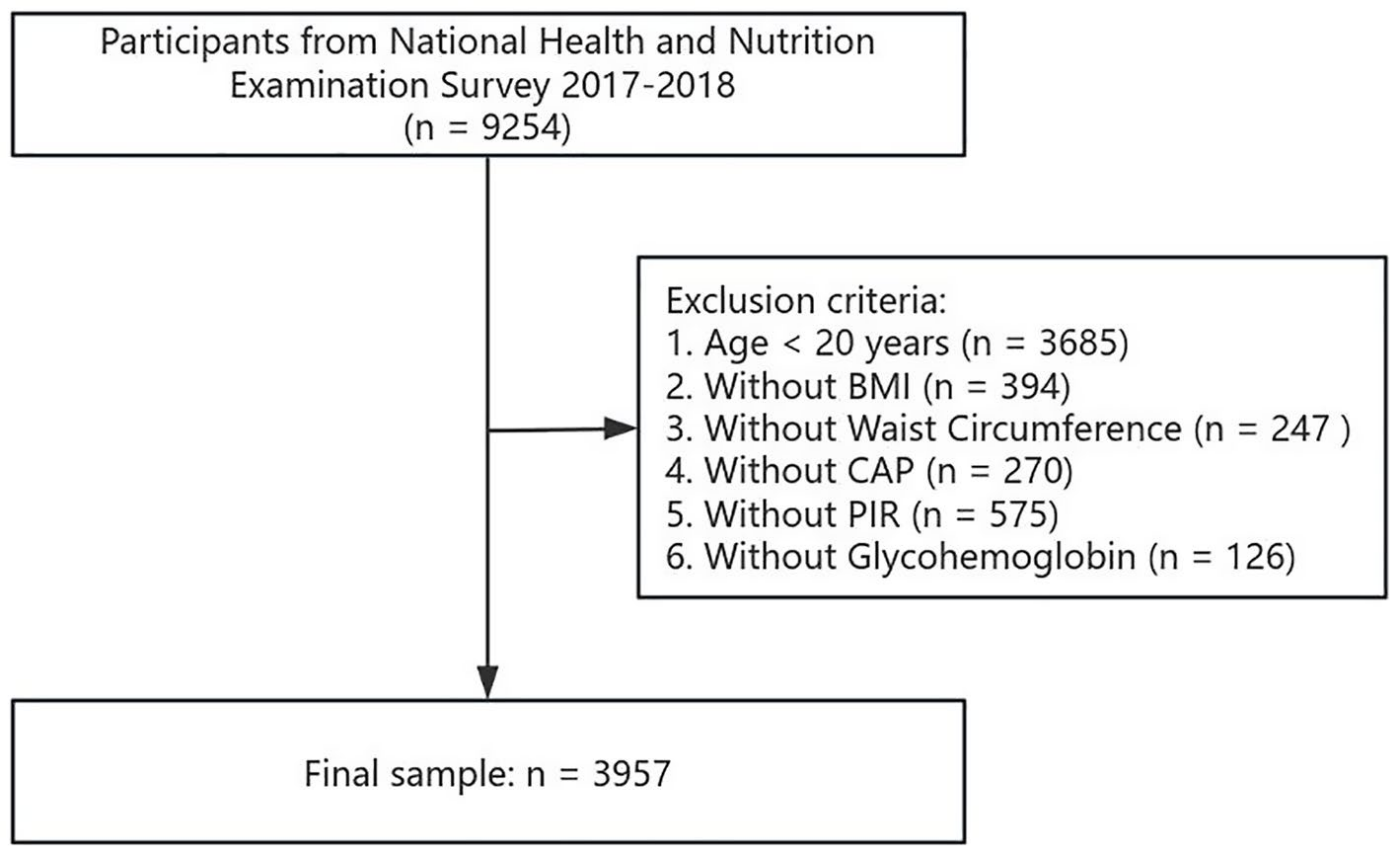
The flow chart of participant selection in the first part of the study.

National Health and Nutrition Examination Survey (NHANES) choose a typical sample of the US non-hospitalized general population using a complex multistage probability sampling design. Vibration-controlled transient elastography was first introduced to estimate hepatic steatosis by controlled attenuation parameters (CAP) during the NHANES 2017–2018 period. This study focused specifically on the 2017–2018 NHANES, which had a total of 9,254 participants. The study was divided into two main parts: the first investigated the link between eGDR and MASLD; the second investigated the link between eGDR and lipids. For the first part, the study eliminated participants who were younger than 20 years old (*n* = 3,685), and those who lacked body mass index (BMI) data (*n* = 394), WC data (*n* = 247), CAP data (*n* = 270), poverty income ratio (PIR) data (*n* = 575) and glycosylated HbA1c data (*n* = 126). The analysis sample for this part finally included 3,957 participants. Details of the screening process are shown in Figure 1.

**Figure 2:**
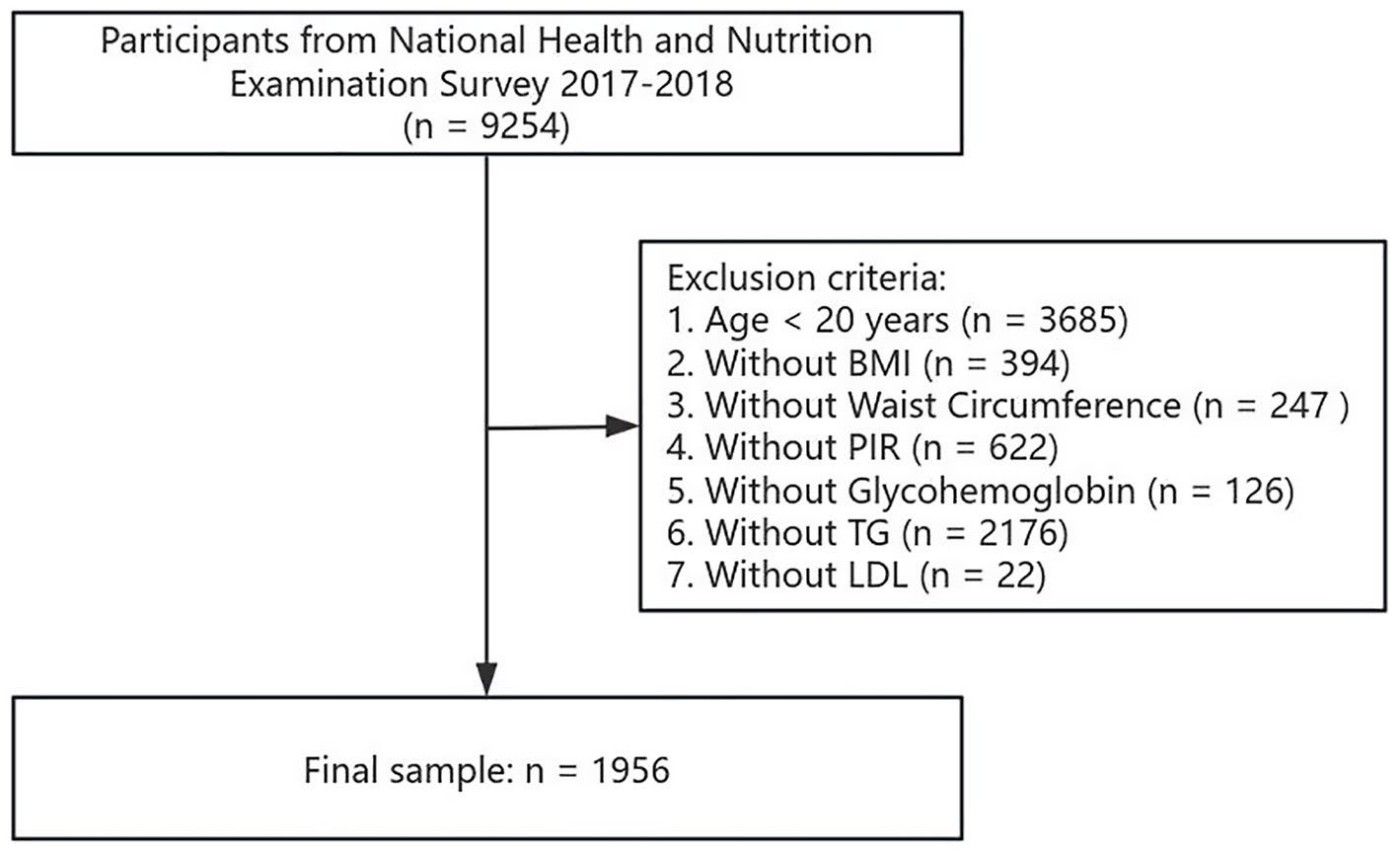
The flow chart of participant selection in the second part of the study.

For the second part, we excluded participants who were under 20 (*n* = 3,685), and those who lacked BMI data (*n* = 394), WC data (*n* = 247), PIR data (*n* = 622), glycosylated HbA1c data (*n* = 126), triglyceride (TG) data (*n* = 2,176), as well as low-density lipoprotein (LDL) data (*n* = 22). The analysis sample for this part ultimately included 1,956 participants. Details of the screening process are shown in Figure 2.

### 2.2 Definition of MASLD

To confirm the diagnosis of MASLD, a CAP score ≥ 285 dB/m must achieved, a threshold with 80% sensitivity and 77% specificity in identifying hepatic steatosis, regardless of other etiology of chronic hepatopathy (10). To be diagnosed, individuals were also required to exhibit a minimum of one cardiometabolic risk factor listed below: overweight/obesity/central obesity, hyperglycemia or diabetes, hypertension, elevated TG, and lower high-density lipoprotein cholesterol (HDL-C), including:

- BMI ≥ 25 kg/m^2^, or WC > 94 cm (men) or 80 cm (women);
- Fasting blood glucose (FBG) ≥ 5.6 mmol/L [100 mg/dL], or glycosylated HbA1c ≥ 5.7% [39 mmol/L], or been diagnosed with diabetes, or diabetes therapy;
- Blood pressure (BP) ≥ 130/85 mm Hg, or taking specific antihypertensive drugs;
- Plasma TG ≥ 1.70 mmol/L [150 mg/dL], or taking lipid-lowering drugs;
- Plasma HDL-C ≤ 1.0 mmol/L [40 mg/dL] (man) and ≤ 1.3 mmol/L [50 mg/dL] (woman), or on lipid-lowering therapy (11, 12).

### 2.3 Definition of eGDR

Estimated glucose disposal rate (mg/kg/min) is an index used to assess IR and is calculated as: eGDR = 21.158 − (0.09 × WC) − (3.407 × HT) − (0.551 × HbA1c) [where WC indicates waist circumference (cm), HT indicates hypertension status (1 for yes, 0 for no), and HbA1c indicates percentage of glycated hemoglobin] (13).

### 2.4 Definition of relative fat mass (RFM)

The RFM is calculated based on WC, height, and sex using the following formula: RFM = 64 − (20 × height/WC) + (12 × sex), where sex = 1 for women and 0 for men (14).

### 2.5 Definition of visceral adiposity index (VAI)

Visceral adiposity index is calculated differently for males and females. For males: VAI = [WC/(39.68 + 1.88 × BMI)] × (TG/1.03) × (1.31/HDL). For females: VAI = [WC/(36.58 + 1.89 × BMI)] × (TG/0.81) × (1.52/HDL) (15).

### 2.6 Definition of uric acid to HDL ratio (UHR)

UHR is calculated by dividing serum uric acid (mg/dL) by HDL-C (mg/dL) (16).

### 2.7 Confounding factors

The study chose confounding factors associated with MASLD according to previous studies. Demographic data was acquired via the NHANES database, including age (in years), sex (male or female), race/ethnicity (including White, Black, Mexican, and other), household PIR, and marital status (having or not having a partner). Educational levels were also assessed (senior high school dropout, high school graduation, and post-secondary education). The information was acquired from the NHANES. BMI was calculated as weight/height^2^. CAP (dB/m) data was derived from inspection data. A body shape index (ABSI) was calculated using the following formula (17): ABSI = WC (m) * height (m) ^ (5/6)/weight (kg) ^ (2/3). Smoking status (yes/no) was obtained from the questionnaire. In addition, the following measures were obtained from laboratory test data: HbA1c (%), total cholesterol (TC) (mg/dL), TG (mg/dL), LDL (mg/dL), and HDL (mg/dL). Diagnosis of hypertension referred to guidelines provided by the Joint National Committee on Prevention, Detection, Evaluation, and Treatment of High Blood Pressure (JNC). The standards for assessing hypertension were: systolic blood pressure ≥ 140 mmHg or DBP-diastolic blood pressure ≥ 90 mmHg, and patients taking antihypertensive drugs during the survey period. Criteria for assessment of diabetes were: diabetes diagnosis by physician, HbA1c ≥ 6.5%, FBG ≥ 126 mg/dL, or receiving diabetes medications and insulin.

### 2.8 Statistical analysis

The study population characteristics were first analyzed separately for the two groups of data. The first part investigated the relationship between eGDR and MASLD. The second part investigated the correlation of eGDR with lipids. The data was grouped into four sets according to quartiles of eGDR. We first analyzed the data of the study population separately to explore the characteristics of population distribution in different eGDR groups and whether there were associations between various variables. Multivariate linear regression models were selected, adjusting for other confounders to reveal true effects. Two models (Model I and Model II) were established, and a smoothed curve fitting was used to reveal the complex relationships between these variables. In addition, subgroup analyses of MASLD and CAP were performed to explore the relationship of these factors with different subgroups, thereby more meticulously understanding their role in different populations. The study used multivariate linear regression models and constructed Models I and II to assess the relationship between lipids and eGDR, controlling for other variables that may influence this relationship. Finally, we also performed a subgroup analysis of lipids to investigate the link between lipids and eGDR in different subgroups in order to more precisely identify the effect of liver function for specific populations affected by lipids. Mediation analysis was conducted using the ‘mediation’ package in R to evaluate the indirect, direct, and total effects of eGDR on MASLD through various lipid-related indicators. This analysis quantified the extent to which RFM, HDL, TG, VAI, and UHR mediated the statistical association between eGDR and MASLD. R4.4.1 language and EmpowerStas were used as statistical software.

## 3 Results

### 3.1 Results of the study population

Three thousand, nine hundred fifty-seven subjects participated in Part 1 and 1956 participated in Part 2. The baseline characteristics are shown in Tables 1, 2. The data was divided into four groups according to quartiles of eGDR. Compared to people in the lower eGDR group, those in the higher eGDR group were younger, suggesting that eGDR may be related to age. In addition, the higher eGDR group had more females, which suggests that eGDR may be associated with gender. People with higher eGDR were also less likely to develop diabetes and hypertension, have lower CAP values, and lower prevalence of MASLD, which is consistent with the clinical significance of eGDR. In terms of lipid levels, people with higher eGDR had lower TC and higher HDL, implying a negative correlation between eGDR and age, CAP, MASLD, TC, and a positive one between eGDR and HDL (*p* < 0.001). Further linear regressions were applied to obtain specific correlation relationships.

**Table 1:** Clinical characteristics of the study population in the eGDR and MASLD quartile subgroups.

Characteristic	eGDR Q1	eGDR Q2	eGDR Q3	eGDR Q4	*P*-value
Age	56.47 ± 14.79	53.99 ± 15.92	46.09 ± 16.00	38.60 ± 14.47	<0.001***
Gender					<0.001***
Male	55.64	48.4	51.66	40.73	
Female	44.36	51.6	48.34	59.27	
Race					<0.001***
Mexican American	6.46	6.76	12.7	7.34	
Non-Hispanic White	70.56	64.07	61.93	63.28	
Non-Hispanic Black	12.7	11.45	8.46	9.48	
Other	10.28	17.73	16.91	19.91	
Education					0.1992
Below high school	10.89	10.06	11.06	8.57	
High School or above	89.11	89.94	88.94	91.43	
Marital					<0.001***
Yes	66.52	62.26	67.33	57.77	
No	33.48	37.74	32.67	42.23	
PIR	3.11 ± 1.63	3.06 ± 1.61	3.10 ± 1.64	3.12 ± 1.66	0.8569
Weight	103.97 ± 21.92	86.11 ± 21.80	86.16 ± 15.80	66.01 ± 10.76	<0.001***
Height	169.48 ± 10.06	167.32 ± 10.64	169.14 ± 9.67	167.08 ± 9.40	<0.001***
BMI	36.19 ± 7.00	30.65 ± 6.78	30.05 ± 4.61	23.61 ± 3.14	<0.001***
ABSI	0.0842 ± 0.0043	0.0825 ± 0.0044	0.0817 ± 0.0043	0.0786 ± 0.0040	<0.001***
Smoke					<0.01**
Yes	12.8	18.12	18.77	18.57	
No	87.2	81.88	81.23	81.43	
DM					<0.001***
Yes	41.33	15.72	6.94	1.03	
No	58.67	84.28	93.06	98.97	
Hypertension					<0.001***
Yes	95.22	70.91	7.27	0	
No	4.78	29.09	92.73	100	
CAP	313.23 ± 56.25	275.14 ± 59.23	264.20 ± 51.13	218.11 ± 43.81	<0.001***
MASLD					<0.001***
No	26.86	58.38	65.42	93.85	
Yes	73.14	41.62	34.58	6.15	

Values for categorical variables are presented as count (%), and values for continuous variables are presented as mean ± SD.

PIR, poverty-to-income ratio; BMI, body mass index; ABSI, A body shape index; DM, Diabetes Mellitus; CAP, controlled attenuation parameter; MASLD, metabolic dysfunction-associated steatotic liver disease; eGDR, estimated glucose disposal rate. Asterisks denote significance levels: **(*P* < 0.01), ***(*P* < 0.001).

**Table 2:** Clinical characteristics of the study population by eGDR and lipid quartile grouping.

Characteristic	eGDR Q1	eGDR Q2	eGDR Q3	eGDR Q4	*P*-value
Age	56.78 ± 15.14	53.16 ± 15.73	45.65 ± 15.92	38.44 ± 14.37	<0.001***
Gender					0.0036
Male	56.82	46.77	48.15	45.86	
Female	43.18	53.23	51.85	54.14	
Race					<0.001***
Mexican American	7.12	6.61	13.63	8.05	
Non-Hispanic White	68.97	64.25	62.17	63.07	
Non-Hispanic Black	13.38	9.92	9.96	9.71	
Other	10.53	19.21	14.24	19.17	
Education					0.0167
Below high school	12.68	10.22	13.27	7.84	
High School or above	87.32	89.78	86.73	92.16	
Marital					0.0244
Yes	64.6	61.02	67.12	58.73	
No	35.4	38.98	32.88	41.27	
PIR	3.02 ± 1.64	2.92 ± 1.61	2.91 ± 1.64	3.23 ± 1.64	0.0036
Weight	104.27 ± 22.64	84.67 ± 19.64	86.16 ± 15.80	66.74 ± 11.01	<0.001***
Height	169.70 ± 9.94	167.25 ± 10.52	169.44 ± 9.88	168.06 ± 9.38	<0.001***
BMI	36.20 ± 7.19	30.25 ± 6.53	29.96 ± 4.70	23.56 ± 2.99	<0.001***
ABSI	0.0842 ± 0.0042	0.0824 ± 0.0046	0.0816 ± 0.0044	0.0788 ± 0.0041	<0.001***
Smoke					0.0023
Yes	12.93	21.82	19.08	15.39	
No	87.07	78.18	80.92	84.61	
DM					<0.001***
Yes	44.72	17.14	7.35	1.23	
No	55.28	82.86	92.65	98.77	
Hypertension					<0.001***
Yes	95.04	71.88	8.09	0	
No	4.96	28.12	91.91	100	
TG	130.22 ± 64.99	113.74 ± 61.51	109.81 ± 67.11	79.84 ± 45.68	<0.001***
LDL	106.77 ± 39.12	114.41 ± 33.33	115.74 ± 31.68	108.34 ± 37.03	<0.001***
HDL	48.54 ± 13.06	54.99 ± 16.00	51.70 ± 13.46	59.86 ± 15.08	<0.001***
TC	181.37 ± 43.63	192.14 ± 38.98	189.39 ± 36.74	184.12 ± 41.26	<0.001***

Values for categorical variables are presented as count (%), and values for continuous variables are presented as mean ± SD.

PIR, poverty-to-income ratio; BMI, body mass index; ABSI, A body shape index; DM, Diabetes Mellitus; TG, triglyceride; TC, total cholesterol; HDL, high-density lipoprotein cholesterol; LDL, low-density lipoprotein cholesterol; eGDR, estimated glucose disposal rate. Asterisks denote significance levels: ***(*P* < 0.001).

### 3.2 Multivariate linear regression of eGDR and MASLD

Model I accounted for variables like age, gender, and ethnicity. Model II accounted for variables like age, gender, ethnicity, marital status, education, hypertension, diabetes, smoking habits, and ABSI. In the unadjusted model, a considerable inverse correlation was noticed between eGDR and MASLD (OR = 0.636, 95% CI: 0.615, 0.658, *p* < 0.001). After adjusting confounders, the OR in Model I was 0.604 (95% CI: 0.580, 0.629, *p* < 0.001) and further decreased to 0.396 (95% CI: 0.320, 0.491, *p* < 0.01) in Model II (Table 3).

**Table 3:** Multifactor linear regression of eGDR and MASLD.

Variable	Non-adjusted model	*P*-value	Model I	*P*-value	Model II	*P*-value
	OR [95% CI]		OR [95% CI]		OR [95% CI]	
eGDR	0.636 (0.615,0.658)	<0.001***	0.604 (0.580,0.629)	<0.001***	0.396 (0.320,0.491)	<0.01**

Model I accounted for variables like age, gender, and ethnicity.

Model II accounted for variables like age, gender, ethnicity, marital status, education, hypertension, diabetes, smoking habits and ABSI. Asterisks denote significance levels: **(*P* < 0.01), ***(*P* < 0.001).

### 3.3 Multivariate linear regression for eGDR and CAP

Model I accounted for variability in age, gender, and ethnicity. In Model II, confounding factors regarding age, sex, race, marital status, education, hypertension, diabetes mellitus, tobacco use, and ABSI were considered for analysis. In the unadjusted model, a significant negative correlation was found between eGDR and CAP values, with a *b* value of −12.837 (95% CI: −13.864, −11.809, *p* < 0.001). In Model I, the relationship between eGDR and CAP values remained significant, with a *b* value of −13.248 (95% CI: −14.444, −12.052, *p* < 0.001). In Model II, the b value was −21.375 (95% CI: −25.828, −16.921, *p* < 0.01). The results revealed a significantly stronger inverse relationship between eGDR and CAP values (Table 4).

**Table 4:** Multifactor linear regression of eGDR and CAP.

Variable	Non-adjusted model	*P*-value	Model I	*P*-value	Model II	*P*-value
	Beta [95% CI]		Beta [95% CI]		Beta [95% CI]	
eGDR	−12.837(−13.864, −11.809)	<0.001***	−13.248(−14.444–12.052)	<0.001***	−21.375(−25.828–16.921)	<0.01**

Model I accounted for variables like age, gender, and ethnicity.

Model II accounted for variables like age, gender, ethnicity, marital status, education, hypertension, diabetes, smoking habits and ABSI. Asterisks denote significance levels: **(*P* < 0.01), ***(*P* < 0.001).

### 3.4 Smoothed curve fitting

**Figure 3:**
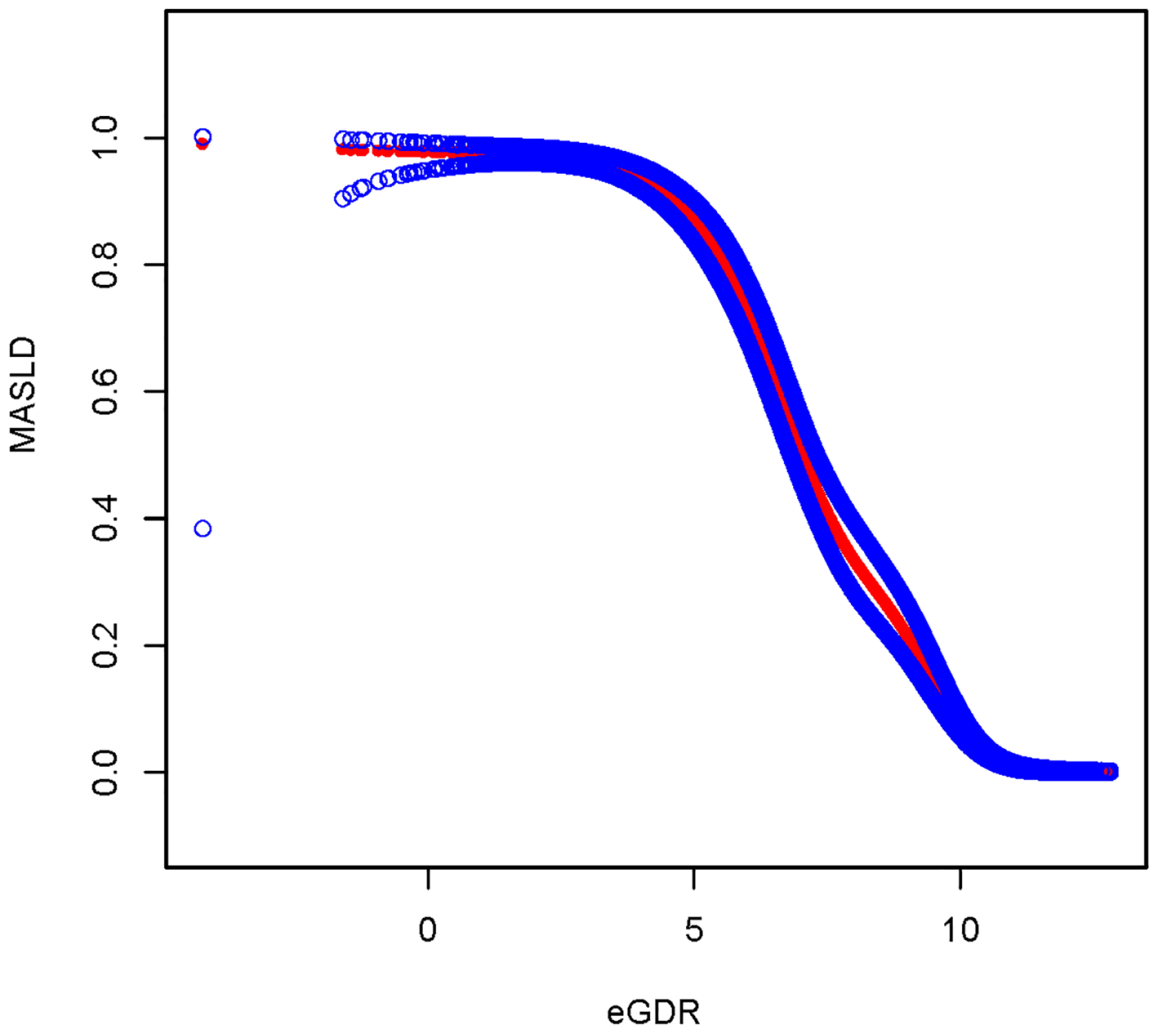
Smooth curve fitting between eGDR and MASLD.

**Figure 4:**
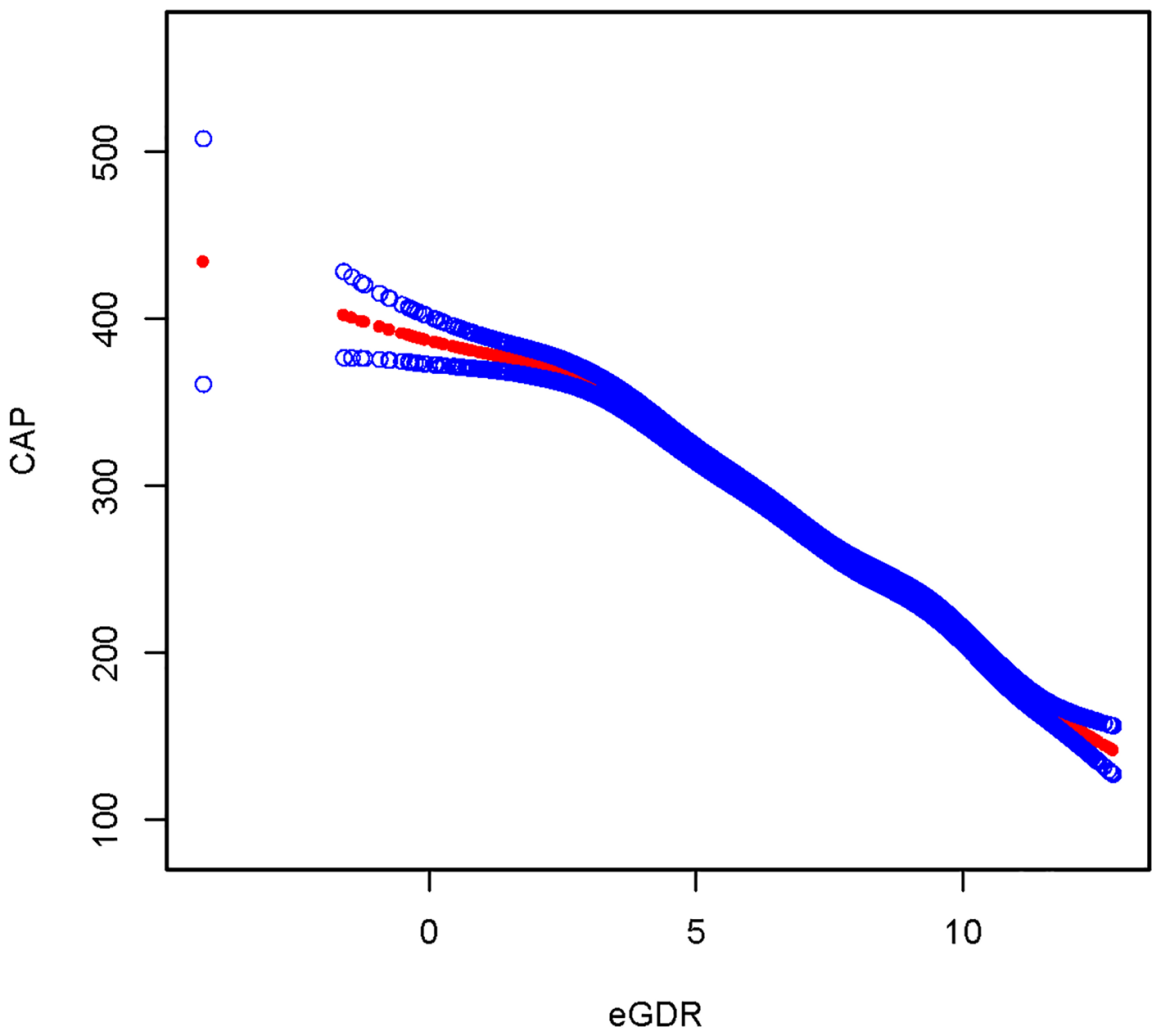
Smooth curve fitting between eGDR and CAP.

After fully adjusting for covariates, the smoothed curve fitting between eGDR and MASLD showed that eGDR exhibited a negative correlation with MASLD, consistent with the results of the multivariate linear regression (Figure 3). The smooth curve fitting between eGDR and CAP revealed that eGDR exhibited a negative correlation with CAP, consistent with the results of the multivariate linear regression between eGDR and CAP (Figure 4).

### 3.5 Subgroup analysis

#### 3.5.1 Subgroup analyses by age

##### 3.5.1.1 Subgroup analysis of eGDR and MASLD by age

It was observed that eGDR had a significant inverse correlation with MASLD in all age groups. In individuals aged 20–39 years, higher eGDR was associated with improved clinical outcomes (i.e., lower risk) with an OR of 0.358 (95% CI: 0.242, 0.532, *p* < 0.01). Similarly, the ORs for eGDR were 0.382 (95% CI: 0.300, 0.487, *p* < 0.01) versus 0.430 (95% CI: 0.299, 0.618, *p* < 0.01) in individuals aged 40–59 years versus those aged 60–80 years, respectively, both of which showed a significant negative correlation. This suggests that clinical outcomes were gradually improved with increased eGDR values among young (20–39 years old), middle-aged (40–59 years old), and older adults (60–80 years old). Notably, these ORs were all significantly lower than 1 (Supplementary Table S1).

##### 3.5.1.2 Subgroup analysis of eGDR and CAP by age

A significant inverse correlation was observed between eGDR and CAP in all age groups. In patients aged 20–39 years, the b-value was −22.816 (95% CI: −27.201, −18.432, *p* < 0.001), indicating that the increase in eGDR in this age group was associated with a significant decrease in CAP. Similarly, *b* value showed significant negative correlations in patients aged 40–59 years (*b* = −19.733, 95% CI: −25.181, −14.285, *p* < 0.01) and in those aged 60–80 years (*b* = −21.577, 95% CI: −27.819, −15.335, *p* < 0.01). The above results further supported the clinical significance of eGDR in different age groups. Especially, in patients aged 20–39 years, the effect of CAP on MASLD was more significant (Supplementary Table S2).

#### 3.5.2 Subgroup analysis by sex

The correlation of eGDR and MASLD was assessed in male versus female populations, showing that eGDR was significantly inversely related to the occurrence of MASLD in both sexes, as shown in Supplementary Table S3. The relationship between eGDR and CAP under gender stratification was further analyzed, which suggested that eGDR was significantly inversely linked with CAP values in both men and women (Supplementary Table S4).

#### 3.5.3 Subgroup analysis by diabetes

The correlation between eGDR and MASLD was assessed in diabetic versus non-diabetic populations, showing a significant relationship in both populations, as shown in Supplementary Table S5. The correlation between eGDR and CAP values in diabetic and non-diabetic patients was further analyzed, which indicated that eGDR and CAP values were significantly negatively correlated in both groups (Supplementary Table S6).

#### 3.5.4 Subgroup analysis based on ethnicity

The association between eGDR and MASLD was evaluated across different ethnic groups, including White, Black, Mexican American, and Other populations. The results demonstrated a significant association in all four groups (Supplementary Table S7). Furthermore, the association between eGDR and CAP values was analyzed within each ethnic subgroup. The results indicated a significant association between eGDR and CAP values across all four ethnic groups (Supplementary Table S8).

### 3.6 Multivariate linear regression of eGDR and lipids

Model I was modified based on age, gender, and ethnicity. Model II was modified based on a variety of factors including age, gender, ethnicity, marital situation, education, presence of hypertension, diabetes, tobacco use, and ABSI. When all covariates were taken into account, the relationship was significant between TG and eGDR (*b* = − 0.005, *p* < 0.05); LDL was not significantly linked with eGDR (*b* = −0.002, *p* = 0.183); HDL was significantly positively linked with eGDR (*b* = 0.036, *p* < 0.01); TC was not significantly associated with eGDR (*b* = 0.0002, *p* = 0.876) (Supplementary Table S9).

### 3.7 Subgroup analysis of lipids

#### 3.7.1 Subgroup analysis by age

The HDL levels were positively correlated with eGDR, especially in patients aged 20–39 years (*b* = 0.045, *p* < 0.01), 40–59 years (*b* = 0.030, *p* < 0.05), and 60–80 years (*b* = 0.024, *p* < 0.05). There was an inverse relation between LDL levels and eGDR in those aged 20–39 years (*b* = −0.007), but not significant (*p* = 0.06). However, LDL did not show a significant correlation with eGDR in individuals aged 40–59 years and 60–80 years (*p* > 0.05). TC levels had no noteworthy correlation with eGDR in any age (*p* > 0.05). TG showed a significant inverse correlation with eGDR in patients aged 20–39 years (*b* = − 0.005, *p* < 0.05), while no consistency was shown in patients aged 40–59 years and 60–80 years (*p* = 0.09 vs. *p* = 0.056) (Supplementary Table S10).

#### 3.7.2 Subgroup analysis by sex

In both male and female groups, eGDR was significantly positively correlated with HDL levels, and increased eGDR may contribute to increased HDL levels. eGDR was significantly negatively correlated with TG in both men and women, suggesting that eGDR may contribute to lowering TG levels, while the effect on LDL and TC was not significant in either sex. Although both sexes showed a significant relationship between eGDR and HDL and TG, females had a stronger inverse relationship between eGDR and TG (larger *b*-value), while males showed a weaker negative correlation in this regard (Supplementary Table S11).

### 3.8 Mediation analysis

Mediation analysis was performed to assess the role of various lipid-related indicators in the association between eGDR and MASLD. The proportions of mediation effects were 61.09% for RFM, 6.79% for HDL, 6.53% for TG, 9.85% for VAI, and 12.9% for UHR. These results are illustrated in Supplementary Figure S1.

## 4 Discussion

The study discussed the link between eGDR and MASLD based on the NHANES database and proved a significant negative correlation. Additionally, the association between eGDR and CAP was investigated. The results demonstrated that eGDR was not only associated with MASLD but also significantly linked to CAP values. By examining disease status as a categorical variable and CAP measurements as continuous variables, the findings further elucidated the association between eGDR and liver health. eGDR is an important measure to assess insulin sensitivity, and lower eGDR often indicates the presence of IR. IR is a key pathophysiological mechanism involved in MASLD (18).

Under normal physiological conditions, insulin regulates blood glucose levels mainly by inhibiting gluconeogenesis in the liver (19), promotes glycogen synthesis (20), and then regulates fatty acid synthesis (21). Despite insulin’s role in managing liver glucose metabolism, such functions are impaired when the body becomes insulin-resistant (22), leading to increased hepatic glucose output (23). At the same time, insulin’s suppression of fatty acid production within the liver may be weakened or possibly intensified, resulting in increased hepatic fat synthesis (24). In addition, IR triggers a series of metabolic disorders that further exacerbate hepatic fat deposition (25). For example, enhanced lipolysis in adipose tissue causes more release of free fatty acids (FFAs) and glycerol (26), which promote gluconeogenesis and lipogenesis after entering the liver (27). At the same time, the decreased ability of skeletal muscle to take up glucose leads to increased blood glucose levels and provides more substrate for hepatic fat synthesis (28).

It has been shown that there are selective changes in the hepatic response to insulin signaling under insulin-resistant conditions. On the one hand, the impairment of glucose metabolism regulation mediated by insulin has been observed; on the other hand, its ability to regulate fatty acid synthesis may not be completely lost or may even continue to promote fat synthesis through novel signaling pathways such as CREBZF (29). This selective IR mechanism may be a major cause of the occurrence and progression of MASLD. Thus, lower eGDR increases the risk of hepatic fat deposition by exacerbating IR, not only affecting hepatic glycometabolism but also facilitating lipogenesis and lipid accumulation within the liver, thereby increasing the likelihood of hepatic steatosis (30, 31). Our subgroup analysis revealed that the association between eGDR and MASLD remained significant in the non-diabetic population. Similarly, Zhang et al. and Peng et al. have also focused on the application of eGDR in non-diabetic populations (9, 32). This process is inversely related to the development and progression of MASLD. By elucidating this mechanism, we can better understand the interplay between glucose metabolism and liver fat deposition, thereby offering novel therapeutic approaches for managing diseases in this realm.

The study also identified a significant inverse relation between eGDR and MASLD. This finding is in agreement with previous studies. For example, Peng et al. further extended this association to liver fibrosis and demonstrated that eGDR exhibited a stronger inverse association with liver fibrosis in patients without diabetes (33). Caprio et al. (34) showed that IR was positively linked with liver fat content in adolescents with obesity, suggesting that IR was a pivotal risk factor for MASLD. Tricò et al. (35) also proved that decreased insulin sensitivity was associated with increased hepatic fat accumulation. In addition, Bonet et al. (36) study noted that adolescents with MASLD exhibited delayed glucose metabolism. Our results are in accordance with former studies and further support the potential value of eGDR in assessing the risk of MASLD.

The study discovered a significant inverse relationship between eGDR and TG, and a notable positive association between eGDR and HDL-C values. This finding suggests that IR is closely linked to the typical dyslipidemia pattern. Specifically, IR increases TG levels by promoting hepatic synthesis and secretion of very low-density lipoprotein. Meanwhile, it lowers HDL-C levels by accelerating their catabolism (37). Peng et al. (33) further proposed a mediating role of the atherogenic index of plasma (AIP). Their findings thus provide insights into the relationship between eGDR and lipid abnormalities. Subgroup analysis of lipids by age showed that the negative association between eGDR and TG was stronger in females. This may be attributed to the role of estrogens in enhancing insulin sensitivity and promoting lipolysis. Subgroup analysis of lipids by age suggests that young people aged 20–39 years should pay more attention to their lipid levels. This finding reveals that the regulatory effect of IR on triglycerides diminishes with age. This may be because younger individuals exhibit more active mitochondrial function and a higher capacity for lipid oxidation (38). This result suggests that insulin sensitivity affects not only hepatic fat deposition but also may influence the risk of cardiovascular diseases by regulating lipid metabolism (39). Therefore, eGDR has potential applications in assessing the overall risk of metabolic diseases.

Mediation analysis results indicate that RFM exhibited the strongest mediation effect among all indicators, accounting for 61.09%. Traditional lipid markers, including HDL (6.79%) and TG (6.53%), showed limited mediation effects. Composite indices, including VAI (9.85%) and UHR (12.9%), demonstrated significantly higher mediation effects than single lipid markers, suggesting that comprehensive assessment of fat distribution (14, 15) or oxidative stress and inflammation (16) provides a more thorough understanding of the association between IR and MASLD. Even after adjusting for these mediators, the direct effect of eGDR on MASLD ranged from 38.91 to 93.47%, indicating that IR may directly drive MASLD through non-lipid pathways.

The results of this research possess significant implications in clinical medicine. First, as an indicator of insulin sensitivity, eGDR is significantly associated with both MASLD and dyslipidemia. In clinical practice, assessing eGDR may aid in identifying risk factors related to these metabolic disorders. Second, this finding also provides ideas for making new therapeutic strategies, such as increasing eGDR levels by improving insulin sensitivity. The study not only reveals the association between eGDR and MASLD, but also further investigates the correlation between eGDR and lipids.

This study has both strengths and limitations. First, This study has both strengths and limitations. First, the NHANES database has become an important resource for metabolic health research worldwide because of its broad population coverage. However, the limitation of NHANES lies in a cross-sectional design of data collection, making it difficult to investigate causality in depth. Nonetheless, NHANES covers broad and diverse populations, which gives it unique advantages in external validity and generalizability. Therefore, in the future, long-term longitudinal studies in different regions and populations are warranted to further validate the clinical utility of eGDR in different populations.

## Data Availability

Publicly available datasets were analyzed in this study. This data can be found: https://www.cdc.gov/nchs/nhanes/.

## Supplementary material

SUPPLEMENTARY FIGURE S1 The graphs in **(A–E)** represented the mediating role of RFM, HDL, TG, VAI and UHR.
