# Supplementary figures and images for: Association between estimated glucose disposal rate and metabolic dysfunction-associated steatotic liver disease and dyslipidemia in US adults: a cross-sectional study
Source: Front Nutr. 2025 Jul 2;12:1621074. doi: 10.3389/fnut.2025.1621074 (PMC12263354; doi:10.3389/fnut.2025.1621074)

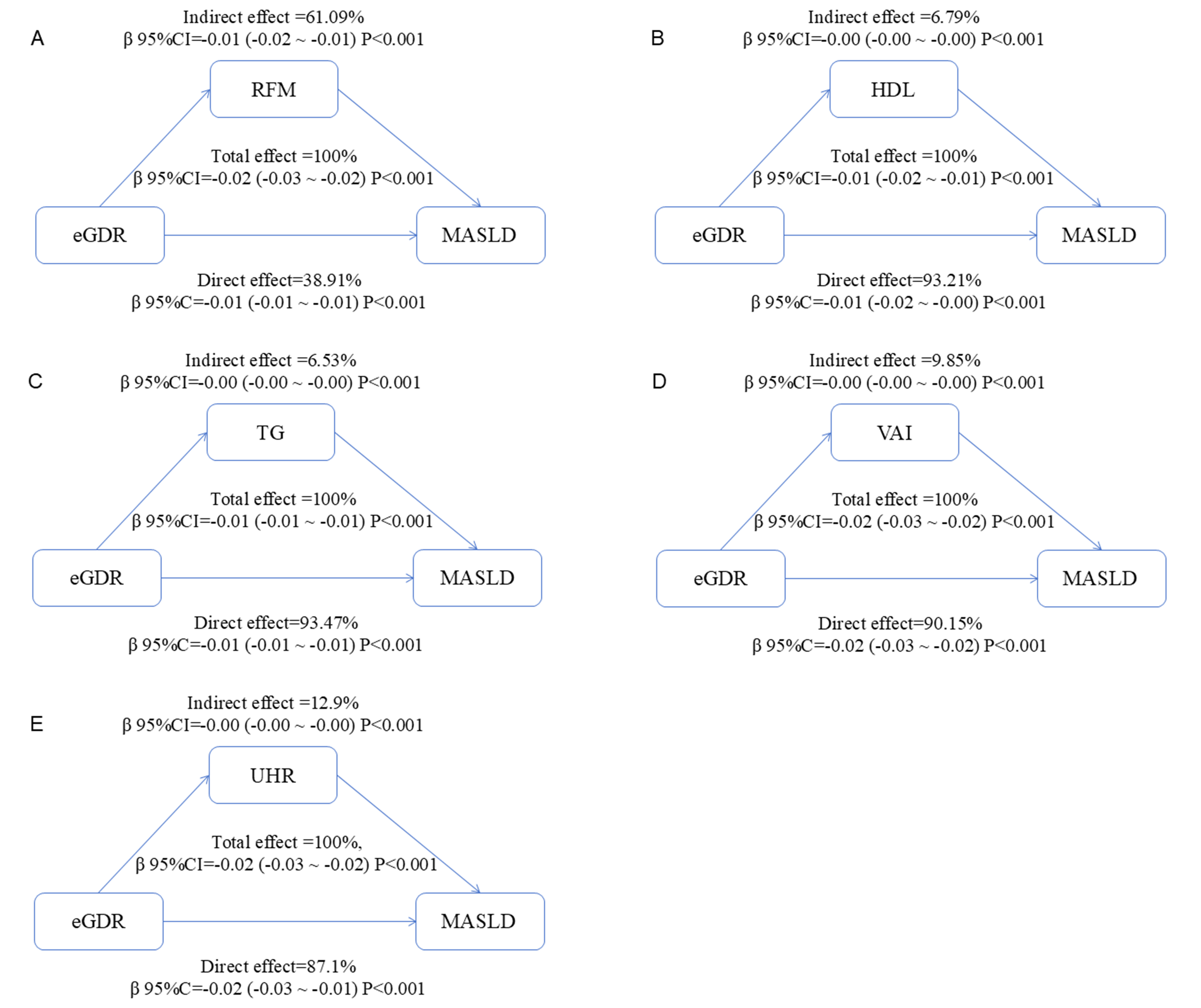

Supplement: SUPPLEMENTARY FIGURE S1 — The graphs in (A–E) represented the mediating role of RFM, HDL, TG, VAI and UHR. [file Image_1.TIF]
