# Supplementary material for: Association between estimated glucose disposal rate and metabolic dysfunction-associated steatotic liver disease and dyslipidemia in US adults: a cross-sectional study
Source: Front Nutr. 2025 Jul 2;12:1621074. doi: 10.3389/fnut.2025.1621074 (PMC12263354; doi:10.3389/fnut.2025.1621074)
Supplement: Supplementary file 2 [file Table_1.DOCX]

Table S1 Age subgroup analysis of eGDR and MASLD

|  | Model II | |
| --- | --- | --- |
|  | OR [95% CI] | P value |
| 20-39 eGDR | 0.358(0.242, 0.532) | <0.01** |
| 40-59 eGDR | 0.382(0.300, 0.487) | <0.01** |
| 60-80 eGDR | 0.430(0.299, 0.618) | <0.01** |

Table S2 Age Subgroup Analysis of eGDR and CAP

|  | Model II | |
| --- | --- | --- |
|  | b [95% CI] | P value |
| 20-39 eGDR | -22.816(-27.201,-18.432) | <0.001*** |
| 40-59 eGDR | -19.733(-25.181,-14.285) | <0.01** |
| 60-80 eGDR | -21.577(-27.819 ,-15.335) | <0.01** |

Table S3 MASLD and eGDR

|  | Model II |  |
| --- | --- | --- |
|  | OR [95% CI] | P value |
| Male eGDR | 0.337(0.260,0.436) | <0.001*** |
| Female eGDR | 0.428(0.319,0.576) | <0.01** |

Table S4 cap values with eGDR

|  | Model II |  |
| --- | --- | --- |
|  | b[95% CI] | P value |
| Male eGDR | -22.619 (-26.688, -18.551) | <0.01*** |
| Female eGDR | -20.370 (-25.347, -15.392) | <0.01*** |

Table S5 MASLD (disease)-eGDR

|  | Model II |  |
| --- | --- | --- |
|  | OR [95% CI] | P value |
| eGDR for people with diabetes | 0.428(0.347 ,0.528) | <0.01** |
| Non-diabetic population eGDR | 0.387(0.321,0.466) | <0.01*** |

Table S6 cap (value) - eGDR

|  | Model II |  |
| --- | --- | --- |
|  | b[95% CI] | P value |
| eGDR for people with diabetes | -17.808(-22.428, -13.188) | <0.01** |
| Non-diabetic population eGDR | -22.075(-25.835, -18.314) | <0.01*** |

Table S7 Race Subgroup analysis of eGDR and MASLD

|  | Model II | |
| --- | --- | --- |
|  | OR [95% CI] | P value |
| Mexican American eGDR | 0.361(0.237,0.551) | <0.001*** |
| Non-Hispanic Black eGDR | 0.467(0.395,0.552) | <0.001*** |
| Non-Hispanic White eGDR | 0.373(0.312,0.446) | <0.001*** |
| Other eGDR | 0.429(0.332,0.554) | <0.001*** |

Table S8 Race Subgroup Analysis of eGDR and CAP

|  | Model II | |
| --- | --- | --- |
|  | b [95% CI] | P value |
| Mexican American eGDR | -21.015(-27.664,-14.366) | <0.001*** |
| Non-Hispanic Black eGDR | -20.941(-23.849,-18.034) | <0.001*** |
| Non-Hispanic White eGDR | -21.752(-25.151,-18.353) | <0.001*** |
| Other eGDR | -20.553(-24.881,-16.225) | <0.001*** |

Table S9 Multifactorial linear regression of eGDR and lipids

|  | Non-adjusted model |  | Model I |  | Model II |  |
| --- | --- | --- | --- | --- | --- | --- |
|  | b [95% CI] | P value | b [95% CI] | P value | b [95% CI] | P value |
| TG | -0.0128(-0.016,-0.009) | <0.001*** | -0.011(-0.014,-0.006) | <0.001*** | -0.005 (-0.008, -0.001) | <0.05* |
| LDL | 0.001 (-0.004, 0.006) | 0.79 | 0.002(-0.002,0.007) | 0.322 | -0.002(-0.007, 0.006) | 0.183 |
| HDL | 0.048 (0.033,0.064) | <0.001*** | 0.056 (0.038 ,0.076) | <0.001*** | 0.036(0.020,0.049) | <0.01** |
| TC | 0.001(-0.004, 0.006) | 0.651 | 0.004( -0.002,0.009) | 0.148 | 0.0002(-0.005, 0.006) | 0.876 |

Table S10 Age Stratification of eGDR and Lipids

|  | Model II |  |
| --- | --- | --- |
|  | b [95% CI] | P value |
| 20-39 eGDR HDL | 0.045 (0.034,0.057) | <0.01** |
| 20-39 eGDR LDL | -0.007 (-0.014,0.001) | 0.06 |
| 20-39 eGDR TC | -0.002 (-0.008,0.005) | 0.48 |
| 20-39 eGDR TG | -0.005 (-0.008,-0.002) | <0.05* |
| 40-59 eGDR HDL | 0.030(0.012,0.048) | <0.05* |
| 40-59 eGDR LDL | 0.003( -0.004, 0.009) | 0.253 |
| 40-59 eGDR TC | 0.004(-0.004, 0.012) | 0.203 |
| 40-59 eGDR TG | -0.004(-0.010 0.001) | 0.09 |
| 60-80 eGDR HDL | 0.024(0.009, 0.038) | <0.05* |
| 60-80 eGDR LDL | 0.001(-0.004, 0.005) | 0.65 |
| 60-80 eGDR TC | 0.002( -0.002 ,0.006) | 0.18 |
| 60-80 eGDR TG | -0.003( -0.006, 0.0001) | 0.056 |

Table S11 Sex Stratification of eGDR and Lipids

|  | Model II |  |
| --- | --- | --- |
|  | b [95% CI] | P value |
| Male eGDR HDL | 0.031(0.019, 0.0426) | <0.01** |
| Male eGDR LDL | -0.003(-0.008,0.001) | 0.112 |
| Male eGDR TC | -0.002(-0.005, 0.002) | 0.219 |
| Male eGDR TG | -0.003(-0.005, -0.001) | <0.05* |
| Female eGDR HDL | 0.034(0.019, 0.050) | <0.01** |
| Female eGDR LDL | -0.001(-0.006 0.004) | 0.476 |
| Female eGDR TC | 0.002(-0.004, 0.007) | 0.477 |
| Female eGDR TG | -0.006(-0.010 -0.003) | <0.05* |
